# Round the Hospitals

**Published:** 1916-12-16

**Authors:** 


### ROUND THE HOSPITALS.

A MEDICAL and surgical nurse confesses perfect ignorance of the benefits to be derived from immediately joining the College Register, and asks us for the great help which a statement of our reasons for the advice we have given to nurses would prove to her and her sisters. She wants to know in what way it will secure or defend the best interests of nurses. The following reasons demonstrate the way:—

**Because** the nurse who registers TO-DAY can do so without submitting herself to further examinations, and once on the Register of the College, she will be on the Legal Register, when the College gets its Register passed by Parliament.

**Because** every nurse who registers and becomes associated with the College of Nursing will obtain a position and status for herself not otherwise to be achieved.

**Because** the College of Nursing will continuously uphold the status of the trained nurse, whilst its Register will protect her from the unfair competition of the untrained and untrustworthy.

**Because** the Registration Bill of the College of Nursing secures the best possible terms for the nurse herself.

**Because** the nurse who now registers and joins the College, at once, is doing her part, by making it possible to obtain the immediate granting of statutory powers by Parliament for the nursing profession and the Registration of Nurses.

**Because** each nurse who omits to register NOW classes herself with those whose policy is to postpone the establishment of any College of Nursing and Nurse Registration indefinitely. Such inaction might possibly deprive trained nurses of both.

**Because** the College of Nursing is incorporated upon a democratic basis, and will, with its Register, be under the control of a Council elected by the nurses themselves.

**Because** the College of Nursing will be the nurses' own College, and if every eligible nurse will now join hands with the *Nursing Mirror*, the nurses can, and will, obtain the organisation and protection they need for themselves and their profession. So the battle of the nurses in their own self-defence can be won.

---

WE are glad to be able to report that our request for the co-operation of the matrons and superintendents throughout the country to aid the *Nursing Mirror* movement to recruit nurses in their own defence by filling up a form of application for registration with the College of Nursing is bearing fruit. We hope that every one of our readers who can help will do so. They will find the scheme fully set out in the *Nursing Mirror* of the 9th instant, page 187, and forms for registration can be obtained from the Editor. The nurses are awakening in increasing numbers to an appreciation of the opportunity they now have, by co-operation and combination, speedily to procure for themselves and their profession, under the direction of their Parliamentary leader, the Hon. Arthur Stanley, M.P., a Royal College and Statutory Recognition. The interest has spread and grows in keenness every week. We are glad to have received inquiries as to the advantages nurses will reap from the College of Nursing and its Register. Some of these advantages are clearly set forth in the left hand column, and we shall be glad to have any criticisms or additions to them that any of our readers may be good enough to supply.

---

A WRITER in *Land and Water* gives an interesting account of a discussion between two Australian men in khaki on the merits of Australian and British hospitals. Here it is: "Some of our outlaws have "run against the discipline of British hospitals and "howled. But give me the British hospital every "time. I have tried both. A nurse in a British hos- "pital is never familiar with an orderly, but in an "Australian hospital they are all on the same level. "It does not work well from the sick man's point "of view. At least that is my experience." The correspondent properly accepts this Australian utterance as evidence that the merits of discipline are rightly appreciated by the best type of Australian. Our experience with regard to Australian patients in hospitals under British administration confirms this view. At first there were difficulties, but they seem to have entirely disappeared, since those responsible for the discipline realised, that, if an Australian is placed upon his honour and treated with confidence, he is one of the most useful, helpful, and easily managed of soldier patients. We should like to have the views of matrons, superintendents, and sisters, and their experience since the war.

---

ONE marked change during the last ten years in American women is their increased alertness and knowledge. It is not uncommon to find gentlewomen of five-and-thirty who have an up-to-date knowledge of affairs, a sound judgment, cultivated powers of speech, an appreciation of the meaning of words, and the capacity to debate questions of moment. Their skill as artists, writers, organisers, architects, disputants, motorists, yachtswomen, and in a multitude of strictly town pursuits and occupations, is remarkable. It has long been the boast that American men make the best husbands, save for the habit of an absence of firmness on occasion. American women, from their education and training, are not infrequently delightful companions and most helpful and admirable friends. The American young lady is seldom shy, and still more seldom at a loss for a word, or the instinct to overcome a difficult position. Is it because of all these good gifts, or in spite of them, that some should be apt to grow selfish?

